# Probiotic Properties and Immunomodulatory Activity of *Lactobacillus* Strains Isolated from Dairy Products

**DOI:** 10.3390/microorganisms9040825

**Published:** 2021-04-13

**Authors:** Luz María Rocha-Ramírez, Ulises Hernández-Chiñas, Silvia Selene Moreno-Guerrero, Arturo Ramírez-Pacheco, Carlos A. Eslava

**Affiliations:** 1 Unidad de Investigación en Enfermedades Infecciosas, Hospital Infantil de México Federico Gómez, Secretaria de Salud, Dr. Márquez No. 162, Col. Doctores, Delegación Cuauhtémoc, Ciudad de México 06720, Mexico; 2 Unidad Periférica de Investigación Básica y Clínica en Enfermedades Infecciosas, Laboratorio de Patogenicidad Bacteriana, Departamento de Salud Pública/División de Investigación, Facultad de Medicina Universidad Nacional Autónoma de México, Unidad de Hemato-Oncología e Investigación, Hospital Infantil de México Federico Gómez/Facultad de Medicina, Universidad Nacional Autónoma de México, Dr. Márquez 162, Col. Doctores, Delegación Cuauhtémoc, Ciudad de México 06720, Mexico; ulisesh@unam.mx (U.H.-C.); eslava@unam.mx (C.A.E.); 3 Departamento de Hemato-Oncología, Hospital Infantil de México Federico Gómez. Dr. Márquez No. 162, Col. Doctores, Delegación Cuauhtémoc, Ciudad de México 06720, Mexico; sswitch@yahoo.com (S.S.M.-G.); artur_tauro@yahoo.com.mx (A.R.-P.)

**Keywords:** Lactobacilli, probiotic, immunomodulation, nitric oxide (NO), antimicrobial activity, interleukin-8 (IL-8), stress inflammatory, adherence inhibition

## Abstract

*Lactobacilli* species are an effective biotherapeutic alternative against bacterial infections and intestinal inflammatory disorders. However, it is important to evaluate their beneficial properties, before considering them as probiotics for medical use. In this study we evaluated some probiotic properties of *Lactobacillus rhamnosus* GG, *Lactobacillus rhamnosus* KLSD, *Lactobacillus helveticus* IMAU70129, and *Lactobacillus casei* IMAU60214 previously isolated from dairy products and as control *Lactobacillus casei* Shirota. Experimental evaluations revealed that all strains expressed hydrophobicity (25–40%), auto-aggregation (55–60%), NaCl tolerance (1–4%), adhesion to Caco-2 cells (25–33%), partial inhibition on adherence of *Escherichia coli* ATCC 35218, *Salmonella* Typhimurium ATCC 14028, and *Staphylococcus aureus* ATCC 23219. Cell-free supernatants (CFS) of *Lactobacilli* also inhibit growth of these pathogens. In immunomodulatory properties a reduction of interleukin-8 (IL-8) and nitric oxide (NO) release was observed in assays with Caco-2 cells stimulated with interleukin-1β (1 ng/mL), or lipopolysaccharide (0.1 µg/mL). On the other hand, the damage induced to Caco-2 cells with sodium dodecyl sulfate (SDS) was attenuated when the cultured cells were pretreated with *L. rhamnosus* KLDS, *L. helveticus* IMAU70129 and *L. casei* IMAU60214. These *Lactobacilli* possess probiotic properties determined by both an antagonistic activity on pathogenic bacteria and reduction in the inflammatory response of cells treated with SDS, a pro-inflammatory stimulant.

## 1. Introduction

The human gastrointestinal tract (GIT) is colonized by different microorganisms known as intestinal microbiota, which establish a symbiotic relationship with their host contributing to its homeostasis [1]. Intestinal microbiota contributes among other factors to intestinal mucosa maturation, functional digestion, metabolic homeostasis, protection against pathogens and immune regulation [2,3]. The microbiota of the GIT belongs to the Firmicutes, Bacteroides, Proteobacteria and Actinobacteria phyla principally and different studies have shown that gut microbiota composition changes are associated with inflammatory bowel diseases (IBD), type II diabetes, rheumatoid arthritis, autism, and Parkinson’s disease [4,5]. Clinical studies have shown positive effects on these alterations when probiotic microorganisms are consumed [5,6]. Probiotic microorganisms that belong to the genus *Lactobacillus* have been reclassified and renamed as genus *Lacticaseibacillus*, which includes *Lacticaseibacillus casei* (*L.casei*) *Lacticaseibacillus rhamnosus (L. rhamnosus)*, and *Lacticaseibacillus specie paracasei* [7]. Species of Lactobacillaceae family as *Lactobacillus acidophilus*, *Lactobacillus helveticus* (*L. helveticus*) *Lactiplantibacillus plantarum)* among others as the well-characterized *Lacticaseibacillus* Shirota (formerly *L*. *casei* strain Shirota), exert a variety of beneficial effects on human health and some of them are considered potential probiotic bacteria [8,9]. Capabilities such as adherence to the epithelium, colonization interference with the colonization of pathogens by production of inhibitory components or competition of sites, and biofilm formation and immunomodulatory activities of intestinal epithelial cells (IEC, are important properties to consider a bacterium as a good probiotic [9,10]. In the IECs there are the sensors and cellular effector components to host defense against intestinal harm [11,12]. Studies with *L. rhamnosus* GG strain (LGG) show that it causesan increase in immunoglobulin A (IgA) producing cells in the intestinal mucosa, interferons are released and there is an improvement in the capture of antigens by lymphoid cells in Peyer’s patches [13]. In induced intestinal inflammatory conditions by sodium dextran sulfate administration, treatment with *L. casei* prevents the inflammation through down-regulation of neutrophil infiltration [14,15]. However, *L. casei* strain Shirota also activates macrophages that produced high interleukin-12 (IL-12) levels, while in chronic inflammatory diseases it exhibits a partial negative deregulation of the secretion of immunological markers [16,17,18]. The effects of *L casei* identified are diverse and can be beneficial or cause harm to the host [19,20]. That is why it is important to evaluate the properties of probiotics to define their potential use as beneficial microorganisms. In a previously study, *L. rhamnosus* GG, *L. rhamnosus* KLDS, *L. helveticus* IMAU70219 and *L. casei* IMAU70214 strains isolated from commercial fermented milk were evaluated showing immunomodulatory properties [21]. However, complete characterization is necessary to define their potential use as probiotic microorganisms. The aim of this study is to characterize *L. rhamnosus* GG, *L. rhamnosus* KLDS, *L. helveticus* IMAU70219 and *L. casei* IMAU70214 by analyzing their physicochemical properties and functional effects on the intestinal mucosa, with the purpose of understanding what cellular mechanisms are activated and their potential effect on health.

## 2. Materials and Methods

### 2.1. Bacterial Strains and Growth Conditions

Five strains of *Lactobacilli* (*L. rhamnosus* GG, *L. rhamnosus* KLDS, *L. helveticus* IMAU70129, *L. casei* IMAU60214 and *L. casei* Shirota) were analyzed. The *Lactobacilli* strains isolated from milk were kindly provided by Dr. Alma Cruz-Guerrero (Universidad Autónoma Metropolitana, Mexico) [22]. The strains were grown on MRS agar (Man, Ragosa and Sarp) and broth culture medium (Franklin Laker, NJ, USA) at 37 °C for 18–24 h. The reference *L. casei* strain Shirota was also grown in MRS broth under the same condition. *Escherichia coli* ATCC 35218, *Salmonella* Typhimurium ATCC 14028, and *Staphylococcus aureus* ATCC 23219 were used as control pathogens. These strains were grown overnight in a tryptic soy broth (TSB) medium at 37 °C. After incubation, the cultures were centrifuged (3000× *g*, 5 min, 4 °C) and washed twice in phosphate-buffered saline (PBS) pH = 7.4. Each bacterial pellet was suspended in sterile PBS at bacteria concentration adjusted to 1 × 10^8^ CFU/mL and used for the different assays. For plating of *Lactobacillus* strains and pathogen bacteria the count was performed with serial dilution (1:100, 1:1000) and 100 μL of the sample was seeded on MRS agar and TSB, respectively. Each test was performed in triplicate sample from three experiments.

### 2.2. Physicochemical Properties of Lactobacillus Strains

#### 2.2.1. Cell Surface Hydrophobicity

The hydrophobicity of the cell surface was evaluated by affinity to organic solvent of the *Lactobacillus* strains in accordance with the method described by Kotzamanidis, et al. [23] with several modifications. Briefly, the bacterial cells were suspended in PBS solution at pH 7.4 to an optical density (OD_600_) of 0.72 corresponding to approximately 10^8^ CFU/mL (A0). A bacterial suspension of 3 mL was mixed with 1 mL of hydrocarbon (xylene). The mixture was shaken in a vortex for 2 min and allowed to stand at 37 °C for 1 h for phase separation. The aqueous phase was gently collected, and the absorbance was measured at 600 nm. The surface hydrophobicity was calculated as:

Hydrophobicity (%) = (1 − A1/A0) × 100,(1)

where A0 is initial absorbance and A1 is final absorbance.

#### 2.2.2. Auto-Aggregation

The auto-aggregative ability of *Lactobacillus* strains was assessed as described previously by Archer, et al. [24] with some modifications. A 4 mL suspension of bacterial cells (10^8^ CFU/mL) was prepared with PBS solution (pH = 7.4) shaken in a vortex for 10 s, followed by incubation without agitation at 37 ℃ during 1, 2, 3, 4, and 5 h. Each time the supernatant was collected, and the absorbance was determined at 600 nm using a quantum spectrophotometer (Amersham Biosciences, Little Chalfont, UK). The auto-aggregation was calculated as:

Auto-aggregation (%) = 1 − (A2/A0) × 100,(2)

where A0 is the initial absorbance at 0 h (OD_600_) of 0.72 (A0) and A2 is the final absorbance at 1, 2, 3, 4, and 5 h of incubation.

### 2.3. NaCl Tolerance Assay

The salt tolerance of the *Lactobacillus* strains was evaluated as described by Archer, et al. [24] with slight modifications. MRS broth with different NaCl concentrations (1–6%) was inoculated with 10 µL of an overnight culture of the *Lactobacillus* strains and incubated at 37 ℃ for 18–24 h. The bacterial growth was then measured by examining the absorbance at 600 nm using a quantum spectrophotometer (Amersham Biosciences, Little Chalfont, UK).

### 2.4. Caco-2 Cells Assay

The *Caco*-2 epithelial cells of a human colon adenocarcinoma (ATCC, HTB-37) were cultured in Dulbecco’s modified Eagle’s minimal essential medium (DMEM; Gibco, Waltham, MA, USA) supplemented with 10% heat-inactivated fetal bovine serum (FBS; Gibco; Waltham, MA, USA) and penicillin (100 U/mL), and streptomycin (100 µg/mL) (Gibco, Waltham, MA, USA). The cells were incubated in a CO2 (5%) oven at 37 ℃. For the adhesion assay, the Caco-2 cells at 2 × 10^5^ cells/mL (final concentration), were deposited in 24-well tissue culture plates (Corning, NY, USA) containing coverslips (1 mm diameter). After the cells became fully differentiated they were maintained in a new DMEM medium 2 h prior to the adhesion assay, subsequently 100 µL with 10^8^ CFU was incorporated in each well of tissue culture plates. The plates were incubated at 37 °C during 3 h under the same conditions specified earlier. To determine the Caco-2 cells adhesion of *Lactobacillus* strains, the cells were methanol (Merck, Darmstadt, Germany) and fixed during 10 min at room temperature. Then, the methanol was completely removed, and the cells were stained with Giemsa (Merck, Darmstadt, Germany) for 20 min at room temperature. To remove the excess stain the coverslips were washed with PBS (pH 7.4). The coverslips were air dried and mounted on glass slides using Entellan (Merck, Darmstadt, Germany). The fields of Caco-2 cells/*Lactobacillus* were observed under an oil immersion microscope at 400× magnification using a Nikon photomicroscope (Nikon Canada Inc., Richmond, BC, Canada).

### 2.5. Quantification of Adherence of Lactobacillus Strains

To determine the number of adhering bacteria, each *Lactobacillus* strain was added to a 1 mL 10^8^ CFU/mL bacterial suspension to Caco-2 cells prepared as indicated in Section 2.4. These plates were incubated for 2 h at 37 ℃ under a 5% CO_2_ atmosphere. The incubation plates with cells were washed four times with a solution sterile of PBS (pH 7.4) to remove the non-adherent bacterial cells. Later the cells were detached by treating them with 100 µL 0.05% TritonX-100 for 5 min at room temperature. Each sample was diluted 10-fold and plated on MRS (Difco Agar, Franklin Lakes, NJ, USA) and incubated for 48 h. Each experimental assay was performed in triplicate. The percent of adhesion was determined by the following calculation:

Adhesion (%) = (V1 × 100)/V0,(3)

where V0 is the initial (CFU/mL) viable count of the tested *Lactobacillus* strains and V1 is the viable bacteria count (CFU/mL) obtained from Caco-2 cells at the end of the experimental assay (i.e., at 2 h after treatment).

### 2.6. Adhesion Inhibition of Pathogen Bacteria by Lactobacillus Strains

This assay was performed using the method described by Jiang, et al. [25] with slight modifications. The Caco-2 cell adherence assay was performed as previously described, but the cells were pretreated with the *Lactobacillus* isolates at 2 h. Later the non-adherent *Lactobacillus* strains were removed with sterile PBS (pH 7.4) washing and then separately the pathogenic bacteria (*E. coli* ATCC 35218, S. Typhimurium ATCC 14028, and *S. aureus* ATCC 23219) were added (ratio 1:1) and incubated for another 1 h. In all cases, non-adherent bacteria were removed as mentioned, and the bacterial counts were determined as described previously in Section 2.1. The counts of the adhered pathogenic bacteria after each assay were calculated as:

% anti-adhesion = (1 − (the number of adherent CFU/mL of pathogenic bacteria pretreated with *Lactobacillus* strains/the number of adhered CFU/mL of pathogenic bacteria in Caco-2 cells non-pretreated with *Lactobacillus* strains)) × 100 (4)

### 2.7. Antimicrobial Activity of Cell-Free Supernatants (CFSs)

The antimicrobial activity of CFSs *Lactobacillus* strains were assessed against *E. coli* ATCC 35218 and *S.* Typhimurium ATCC 14028 Gram-negative bacteria and *S*. *aureus* ATCC 23219 Gram-positive bacteria using the modified agar well diffusion method described by Wang, et al. [26] with slight modifications. The bacterial inoculum was adjusted to 0.5 McFarland tube (1.5 × 10^8^ CFU/mL) and employed as inoculum in Mueller-Hinton agar plates (Difco Agar, Franklin Lakes, NJ, USA). The plates of 7 mm diameter wells were then filled with 100 µL of CFSs and incubated for 10 min at room temperature to allow diffusion into the agar, followed by incubation at 35 °C for 24 h. The antimicrobial activity of each *Lactobacillus* CFSs isolate was evaluated measuring the bacterial inhibition zones. The negative controls were MRS medium (alone) and MRS medium supplemented with tetracycline antibiotic as the positive control.

#### Preparation CFSs from *Lactobacillus* Strains

For the preparation of (CFSs), the 1% (*v*/*v*) inoculum of each *Lactobacillus* isolate was cultured in MRS broth medium and incubated at 37 ℃ for 48 h. The cells were separated by centrifugation at 5000 rpm for 15 min and the supernatant was collected and filtered through a sterilized 0.22 µm Millex-GV filter (Millipore, MA, USA). The CFS was adjusted to a pH of 6.5 with a sterile solution of 1 M sodium hydroxide (NaOH). Finally, the CFSs were stored in 500 µL aliquots at 4 °C.

### 2.8. Immunomodulatory Activitie of Lactobacillus Strains on the Secretion of IL-8 and NO-Induced by LPS on Caco-2 Cells

The protection effect of *Lactobacillus* strains against inflammation mediators (IL-8 and NO) of Caco-2 cells stimulated with 1 ng/mL IL-1β (Pharmingen, NJ, USA) or 0.1 µg/mL LPS of *E. coli* 0111:B4 for 24 h was evaluated measuring the IL-8 levels by ELISA assay kit (Pharmingen, NJ, USA) under basal conditions, with stimulated or without the *Lactobacillus* strains stimulus (ratio 1:1) after 24 h. The NO production levels were quantified by the Griess reaction in accordance with methods described by Archer, et al. [24]. The accumulation of nitrites, as a measure of the secretion of NO, was determined in the cell culture supernatant reading the absorbance at 570 nm using a microplate reader Fluoroskan Ascent FL (Thermo Fisher Scientific, Waltham, MA, USA). The culture medium alone was employed as the blank control.

### 2.9. Inflammatory Stress Protection Induced by Lactobacillus Strains

The protection effect of *Lactobacillus* strains on inflammatory stress was evaluated by Presti, et al. [27]. The inflammatory stress on Caco-2 cells was induced with SDS (Sigma, Darmstadt, Germany) at a concentration of 0.05% prepared in DMEM. Each one of the *Lactobacillus* strains was added to the wells at a concentration of 10^7^ CFU/mL following incubation of 24 h. The cell viability was evaluated using the 3- (4,5-bromide dimethylthiazol-2-yl) -2,5-diphenyltetrazole (MTT) assay (Sigma, Darmstadt, Germany) at a concentration of 0.05% MTT in DMEM, followed by incubation for 4 h at 37 °C under a 5% CO_2_ atmosphere in the dark. After the medium was removed, 100 µL of dimethyl sulfoxide (DMSO; Sigma, Darmstadt, Germany) was added to each well and the reduction of MTT was measured at an absorbance of 570 nm using a microplate reader Fluoroskan Ascent FL (Thermo Fisher Scientific, Waltham, MA, USA) with DMSO alone serving as the blank control.

### 2.10. Statistical Analysis

The results are expressed as the mean ± standard deviation (SD) of three independent experiments. The statistical analysis was performed using the GraphPad Prism 5 (San Diego, CA, USA). The obtained data were subjected to one- or two-way analysis of variance (ANOVA) and *p* < 0.05 was considered statistically significant.

## 3. Results

### 3.1. Physicochemical Properties of Lactobacillus Strains

#### 3.1.1. Cell Surface Hydrophobicity

**Figure 1:**
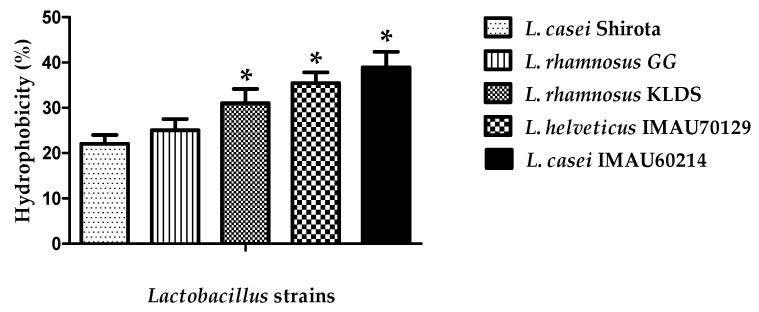
*Lactobacillus* strain hydrophobicity determined in xylene presence (1 h). Values are expressed as mean ± standard deviation (SD) in three different experiments. * significant difference among strains *p* < 0.05.

The *Lactobacillus* strains displayed different values of hydrophobicity (Figure 1). *L. rhamnosus* KLDS, *L. helveticus* IMAU70129, and *L. casei* IMAU60214 showed a significant moderate hydrophobicity (31 ± 3.1%, 35.4 ± 2.4%, and 38.9 ± 3.4%), respectively after treatment with xylene. In contrast, lower values were observed for both *L. rhamnosus* GG (25 ± 2.0%) and *Lacticaseibacillus* strain Shirota (22 ± 2.5%), which was used as reference.

#### 3.1.2. Auto-Aggregation

**Figure 2:**
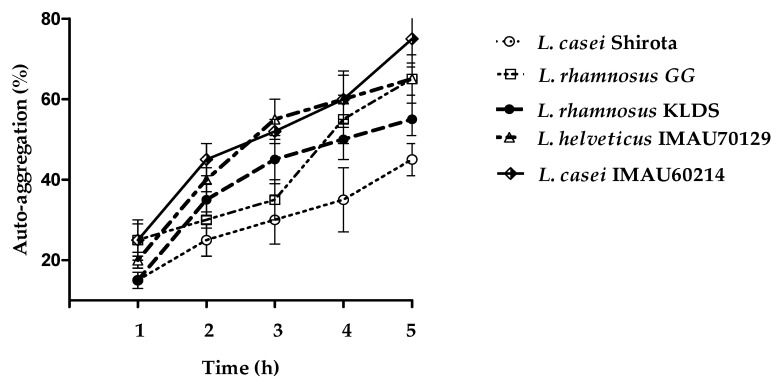
Auto-aggregation of *Lactobacillus* strains as determined after various times of incubation durations at 37 °C. The values represent the mean value ± standard deviation (SD) of three different assays.

The auto-aggregation properties of the evaluated *Lactobacillus* strains showed variable scales which were time dependent (Figure 2). The analysis of *L. rhamnosus* KLDS, *L. helveticus* IMAU70129, and *L. casei* IMAU60214 showed high auto-aggregation levels to early time (3 h), while *L. rhamnosus* GG and *L. casei* Shirota showed lower activity in early incubation times which gradually increased after 5 h.

### 3.2. NaCL Tolerance

**Figure 3:**
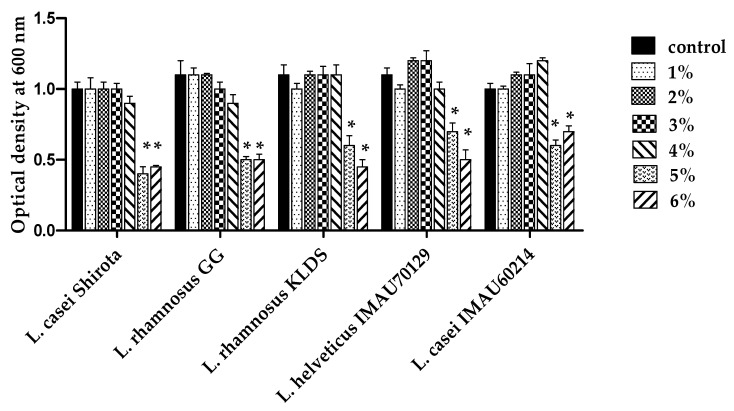
NaCl tolerance of *Lactobacillus* strains. The tolerance to different salt concentrate ions was analyzed during 18 h at 37 °C. Values represent the mean ± standard deviation (SD) of three different assays. An MRS medium without NaCl was used as the control. * significant difference among strains at concentration of NaCl-MRS.

To investigate the effect of high concentrations of salt on growth, the *Lactobacillus* strains were cultured in MRS medium supplement with different NaCl concentration during 18 h. The observed results showed tolerance of *Lactobacillus* strains studied defined by the growth of bacteria to 1–4% NaCl concentrations (Figure 3).

### 3.3. Caco-2 Cells Adherence Assay

**Figure 4:**
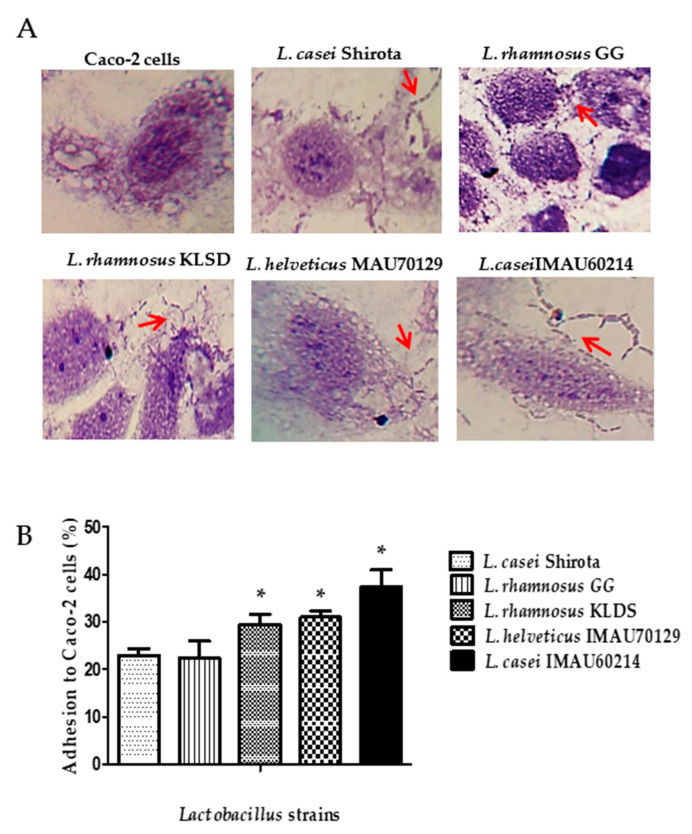
Adhesion property of *Lactobacillus* strains to Caco-2 cells. (**A**) Representative microscopic visualization (400×). The red arrow indicates the evaluated *Lactobacillus* strains adhered to Caco-2 cells and the index score (number of *Lactobacillus* per Caco-2 cells). (**B**) Percentage of adhesion on Caco-2 cells. The values represent the mean ± SD of three separated experiments. * significant difference among strains (*p* < 0.05).

The qualitative test to determine the adherence capacity of *L. rhamnosus* GG, *L. rhamnosus* KLDS, *L. helveticus* IMAU70129, and *L. casei* IMAU60214 used the human epithelial Caco-2 cell line. This test showed the *Lactobacillus* strains adhere to cells after two hours of incubation (Figure 4A). The quantitative analysis report values varied from 25% to 35%. *L. rhamnosus* KLDS, *L. helveticus* IMAU70129, and *L. casei* IMAU60214 had higher adhesion, compared with *L. casei* strain Shirota (Figure 4B).

### 3.4. Adherence Inhibition of Pathogen Bacteria by Lactobacillus Strains

**Figure 5:**
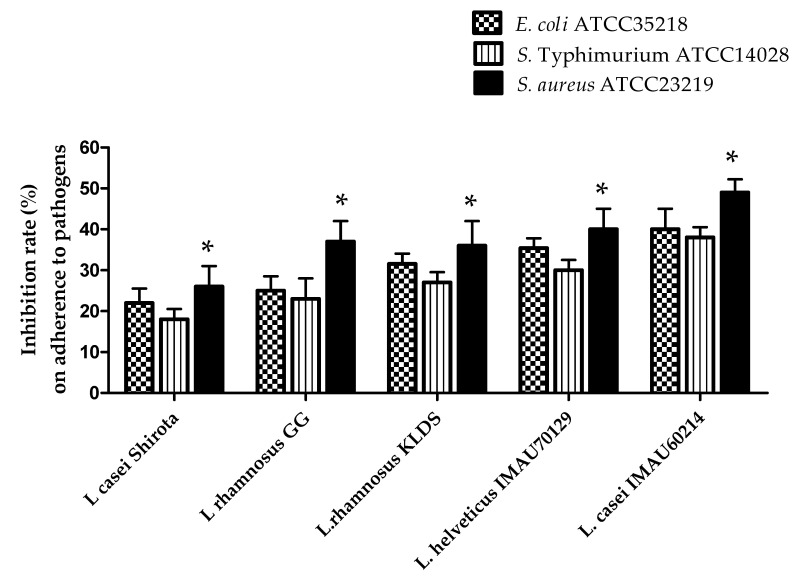
Adherence inhibition effect of *Lactobacillus* strains against pathogenic bacteria as well as *Escherichia coli* ATCC 35218 and *Salmonella Typhimurium* ATCC 40128 and *Staphylococcus aureus* ATCC 23219. The values represent the mean ± SD of three different assays. * significant among *Lactobacillus* strains (*p* < 0.05).

*Lactobacillus* strains were evaluated for their ability to interfere with the adhesion of human pathogens on epithelial Caco-2 cells. The results showed that some of the *Lactobacillus* strains evaluated induced a significant decrease in the adherence of pathogens to Caco-2 cells when compared with the reference probiotic *L. casei* strain Shirota (Figure 5). *L*. *rhamnosus* KLSD, *L*. *helveticus* IMAU70129, and *L. casei* IMAU60214 exhibited anti-adhesion effect against *E. coli* (30 ± 2.5%), *S. Typhimurium* (45 ± 3.5%) and *S. aureus* (50 ± 2.5%) respectively. In contrast, the anti-adhesion values observed with *L. rhamnosus* GG (25 ± 2.5%) and the reference probiotic *L. casei* strain Shirota (27 ± 3.1%) showed less activity.

### 3.5. Antimicrobial Activity of Cell-Free Supernatants (CFSs) from Lactobacillus Strains

**Figure 6:**
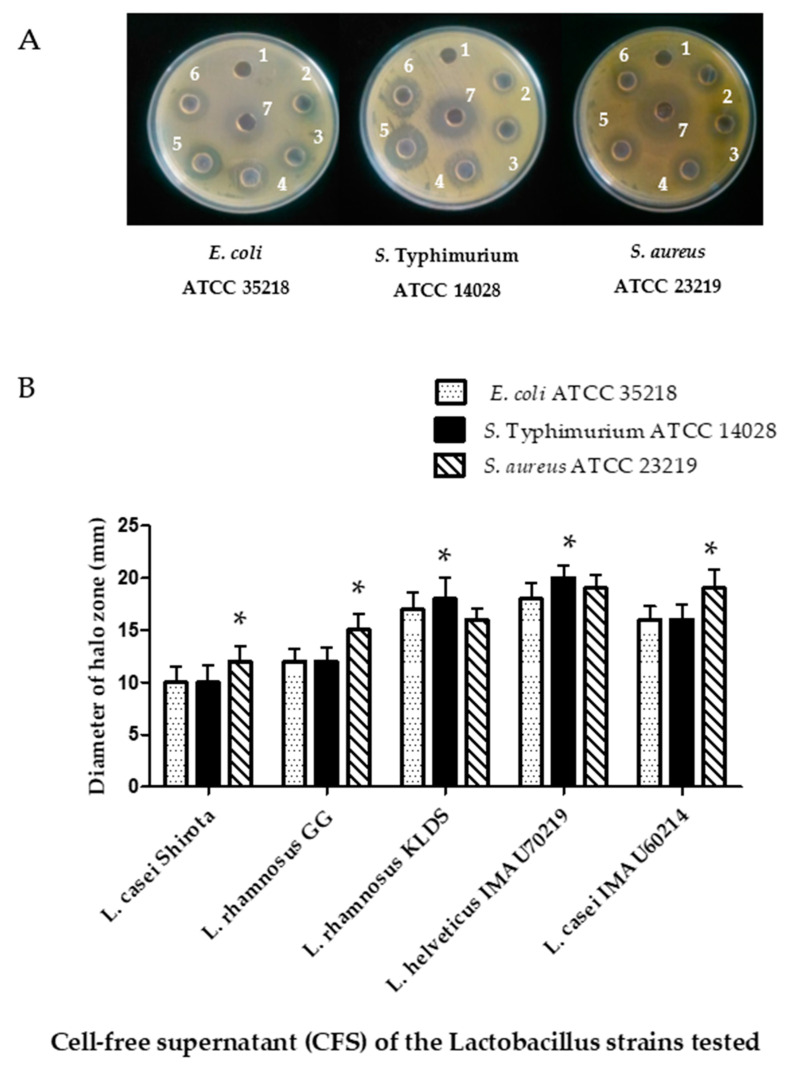
Antimicrobial activity of cell-free supernatant (CFS) of *Lactobacillus* strains against pathogenic bacteria. (**A**) Well 1, control MRS (alone) or negative (not-inhibitory); Well 2, *L. rhamnosus* GG; Well 3, *L*. *rhamnosus* KLDS; Well 4, *L*. *helveticus* IMAU70129; Well 5, *L. casei* IMAU60214; Well 6, *L. casei* strain Shirota; Well 7, MRS with tetracycline antibiotic (positive showed inhibitory activity). (**B**) Zone inhibition in diameter (mm). Values represent the mean ± SD of three different assays. * significant difference among *Lactobacillus* strains (*p* < 0.05).

The obtained results in this assay shown that the cell-free supernatants tested inhibited the growing pathogenic bacteria evaluated (Figure 6A). The highest inhibition zone was recorded against *S. aureus* ATCC 29213 (16 mm), followed by S. Typhimurium ATCC 14028 (10 mm) and *E. coli* ATCC 35218 (12 mm) after 24 h of incubation (Figure 6B). *L. helveticus* IMAU70129, *L. casei* IMAU60214, and the reference probiotic *L. casei* strain Shirota showed higher inhibition zones than *L. rhamnosus* GG.

### 3.6. Immunomodulatory Activity of Lactobacillus Strains on the Secretion of IL-8 and NO-Induced by LPS on Caco-2 Cells

**Figure 7:**
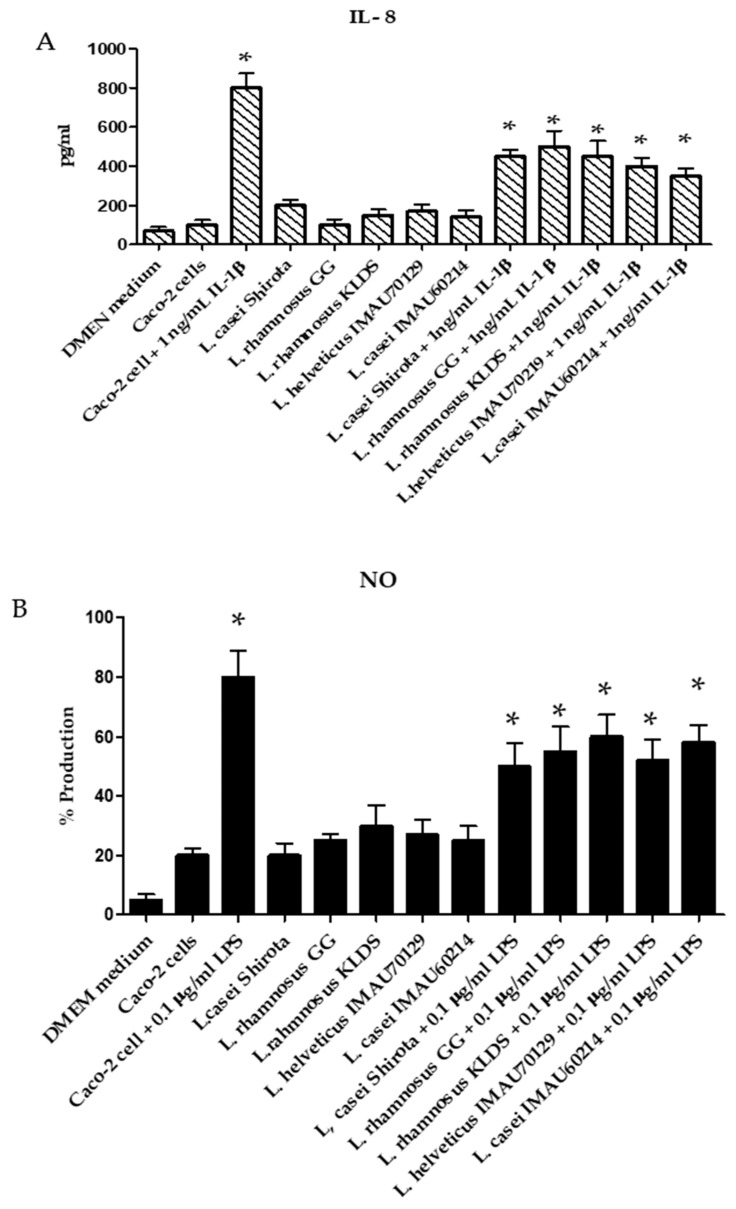
Immunomodulatory activity of *Lactobacillus* strains on the secretion of interleukin-8 (IL)-8 and NO on Caco-2 cells. (**A**) Baseline IL-8 response and production in an IL-1β (1 ng/mL) stimulated inflammatory environment. (**B**) Nitric oxide production at baseline and under an inflammatory environment stimulated with LPS (0.1 µg/mL). Values represent the mean ± SD (*n* = 3). * significant differences among *Lactobacillus* strains (*p* < 0.05).

A feature to consider for a potential probiotic microorganism is its property of immunomodulation of the intestinal epithelium. An assay with Caco-2 cells incubated during 12 h with *Lactobacillus* strains induced a slight increase of IL-8 levels (250 ± 13 pg/mL), like those observed on unstimulated epithelial cells (150 ± 10 pg/mL). On the other hand, the incubation of Caco-2 cells with IL-1β (1 ng/mL) or LPS (0.1 µg/mL) significantly increased the IL-8 secretion levels (800 ± 15 pg/mL) (Figure 7). However, prior to stimulation of Caco-2 cells with IL-1β (1 ng/mL) or LPS (0.1 µg/mL), the cells were incubated with *L. rhamnosus* KLDS, *L. helveticus* IMAU70129, and *L. casei* IMAU60214 strains and a reduced secretion of IL-8 was observed (Figure 7A). was Another interesting observation was that stimulation with LPS of *E. coli* 011:B4 (0.1 µg/mL) of Caco-2 cells previously incubated for 12 h with *L. rhamnosus* KLDS, *L. helveticus* IMAU70129, and *L. casei* IMAU60214 inhibited NO production (Figure 7B).

### 3.7. Inflammatory Stress Protection Induced by Lactobacillus Strains

**Figure 8:**
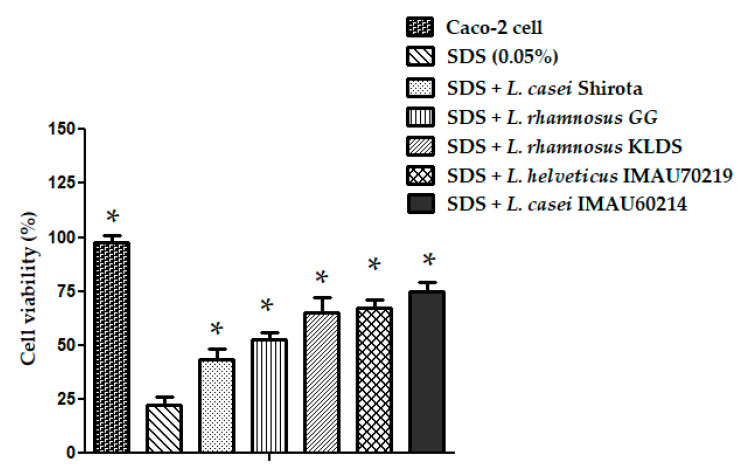
Protective effect on Caco-2 cells induced by *Lactobacillus* strains. The residual cell viability was measured by the mean MTT assay. The results are expressed in percentages. Values represent the mean ± SD of three separated assays. * significant differences among *Lactobacillus* strains versus SDS 0.05% (*p* < 0.05).

The in vitro inflammatory stress model of human epithelial Caco-2 cells exposed to SDS was used to evaluate the potential protective effect of *Lactobacillus* strains (*L. rhamnosus* GG, *L. rhamnosus* KLDS, *L. helveticus* IMAU70129, and *L. casei* IMAU60214). Co-incubation of Caco-2 cells with *Lactobacillus* strains for 24 h resulted in a significant reduction in the damage produced by SDS exposition (0.05%) compared with the untreated Caco-2 cells. In the assay L. *rhamnosus* KLDS, *L. helveticus* IMAU70129, and *L. casei* IMAU60214 exhibited a greater viability percent of the Caco-2 cells when compared to the cells only exposed to SDS (Figure 8).

## 4. Discussion

Human colonization by bacteria occurs at childbirth, as well as through breastfeeding and by environmental microorganism. Through breast milk the infant acquires different microorganisms, mainly *Lactobacilli* and bifidobacteria. The symbiotic relationship between human and microbiota contributes to establish a partnership that benefits both. The microorganisms acquire nutrients and a niche to reproduce and live while the host receives substrates that it is not capable of producing. In this way, it is protected against colonization by pathogenic microorganisms and the immune system matures [28]. Changes in the composition and structure of the intestinal microbiota have been linked to diseases such as diabetes, inflammatory syndromes such as ulcerative colitis, and neurological alterations like autism and Parkinson’s [29,30]. Changes in the intestinal microbiota have been related to diet and the inappropriate use of antimicrobials [2,8]. When microbiota alterations occur, the use of probiotic microorganisms is proposed. However, it is necessary to evaluate the specific characteristics and safety of the probiotic bacteria before using them to avoid causing damage to the host [31,32].

In this study, we evaluated different properties of the potential probiotic *L rhamnosus* KLDS, *L. helveticus* IMAU70129, and *L. casei* IMAU60214, which were isolated from commercial fermented products. Hydrophobicity, auto-aggregation, cell adhesion, antagonistic activity against enteric pathogens and immunomodulatory effects on Caco-2 cells were evaluated. The cell-surface hydrophobicity and cell-surface charge of bacteria are recognized as physicochemical variables for evaluating bacterial adhesion to surfaces [33]. In this study we observed that the hydrophobicity values of the *Lactobacillus* strains were similar to those observed in other probiotic strains reported elsewhere [34,35]. The hydrophobicity results (30–50%) support that the studied strains could be classified as having moderate hydrophobicity in accordance with the report by Jiang, et al. [25]. The hydrophobic characteristics of a strain have been implicated in bacteria adhesion to host tissues, conferring a competitive advantage on pathogen bacteria toward maintaining homeostasis in the host’s GIT [15,36,37].

Adherence between the same bacteria (auto-aggregation) helps bacteria adhere to the surface of the intestinal mucosa so that it persists in the intestine. Consequently, it competes with pathogenic bacteria and interferes with their establishment in the GIT to promote host health [38,39,40,41]. In the study the auto-aggregation results show that in *L. casei* IMAU60214 this property was very similar with what was observed in *L. rhamnosus* KLDS and *L. helveticus* IMAU70129.

Auto-aggregation and hydrophobicity are properties utilized as selective screening of potential probiotic strains. However, there is controversy when both properties correlate with epithelium adherence [42,43,44,45]. In this study, the cell adherence to Caco-2 cells, auto-aggregation, and hydrophobicity of the evaluated *Lactobacillus* strains was positive but with some differences between each other and with what we observed in the *L. casei Shirota* reference strain. This could be due to the difference in responses between strains which ultimately contributes to the bacteria interacting with the epithelium of their potential host. The *Lactobacillus* adherence initially could be associated with hydrophobic interactions and later specifically associated throughout bacterial structures as pilis which interact with cell receptors [46]. The identification and characterization of bacteria structures is of great importance in probiotic strain studies, since this would facilitate the study of the mechanisms associated with the beneficial properties of these microorganisms.

As previously mentioned, many different characteristics must be considered when selecting potential probiotic microorganisms. In addition to adherence, other properties need to be analyzed. Different studies have reported that several groups of probiotic strains can be protective, especially through interfering with the adherence and colonization of pathogenic bacteria [47,48]. In this study, anti-adhesion activity against pathogenic strains was analyzed (*E. coli* ATCC 35218; *S.* Typhimurium ATCC 12840 and *S. aureus* ATCC 23219). The results show interference of the pathogen bacteria (30–35% inhibition of adhered bacteria), similar to what was reported in studies performed with *L. paracasei* strains [49,50]. Although *S. aureus* does not act directly on the intestinal epithelium since it is through its enterotoxin, we evaluate the interference effect of *Lactobacillus* strains on this bacterium because dairy products can be enriched with probiotics and in this way they prevent staphylococci contamination.

The probiotic strains can also antagonize with pathogen bacteria both by antimicrobial activity mediated by metabolism secreted molecules or through immunomodulatory activities of the host’s immune system [9,12]. In this study, different growth inhibition of the selected pathogens induced by secreted products of *Lactobacillus* strains were observed. *L. casei* IMAU60214 and *L. helveticus* IMAU70129 show a more intense activity than the other studied strains, which was determined by the size of growth inhibition halos. Previous studies with *L. reuteri* isolated from humans show moderate inhibition against *E. coli* (inhibition of 7–10 mm in diameter) [50]. This is in contrast to what has been observed with the *L. delbrueckii* subspecies *bulgaricus* which exerted a substantial inhibition (21.1 mm in diameter) against *E. coli* [51]. It is also in contrast to what has been observed with *L. plantarum* B7 isolated from dyspeptic patients which induced moderate growth inhibition of *H. pylori* (11–13 mm) [52]. The differences in the bacterial growth inhibition effects observed in our study could be attributed to acidic products such as lactic and acetic acids, or compounds like hydrogen peroxide, ethanol, diacetyl, acetaldehyde, acetoin, reuterin, reutericyclin, and bacteriocins, among others. However, in the future it will be necessary to characterize the components responsible for these effects at the molecular level.

The immunomodulatory effect of probiotics has been considered as other one of the potential clinical effects of these microorganisms. This is why the term Bio-immunobiotics has been proposed to designate probiotic microorganisms with the ability to modulate the immune response [53]. In this study some immunomodulatory properties of the *Lactobacillus* strains analyzed were identified. One of these was the diminishing effect in the production of IL-8, similar to results observed with *L. plantarum* in previous studies [54,55]. The oxidative attenuation of NO that induces cytotoxic mechanisms under oxidative stress conditions was also evaluated. *L. rhamnosus* GG, *L. helveticus* IMAU70129, and *L. casei* IMAU60214 showed protective abilities against damage caused by SDS. This result is important because oxidative stress conditions can be extremely harmful to the epithelium, which suggests that these strains can contribute to reducing the inflammatory processes in the mucosa and may play a functional role in the prevention and control of several acute and chronic infectious diseases. Our research findings allow us to define these lactobacillus strains as probiotic candidates for the first time. However, the main limitation of the study is that the results were obtained by in vitro tests where the challenge and response conditions are controlled. Therefore, it is necessary to carry out studies in animal models and in humans to explore their safe use and explore the probable mechanisms of clinical application of these strains.

## 5. Conclusions

Studies of the gut microbiota on health and disease are of high importance, and the role of probiotics and prebiotics as modulators of the microbiota require studies to find the best candidates. The obtained results show that the *Lactobacillus* strains evaluated possess properties such as adherence to cultured cells, inhibition of the colonization and growth of pathogenic bacteria, and regulation of the inflammatory response. All of these are elements that make them good candidates for use as probiotics. However, it is important to evaluate their properties in animal models to ensure they are safe to use in clinical applications.

## Data Availability

The data presented in this study are available on request from the corresponding author.
